# A Molecular (Not Very Becoming) Picture of Stressed Arteries and Heart, with Some Therapeutic Hope

**DOI:** 10.3390/ijms24043870

**Published:** 2023-02-15

**Authors:** Maria G. Barderas, Fernando de la Cuesta

**Affiliations:** 1 Department of Vascular Physiopathology, Hospital Nacional de Parapléjicos (HNP), SESCAM, 45071 Toledo, Spain; 2 Department of Pharmacology and Therapeutics, School of Medicine, Universidad Autónoma de Madrid, 28029 Madrid, Spain; 3 Instituto de Investigación Sanitaria del Hospital Universitario La Paz (IdiPAZ), 28029 Madrid, Spain

This Special Issue has focused on molecular mechanisms (vascular calcification, endothelial dysfunction, cardiac remodelling, inflammation, oxidative stress, etc.) and pathophysiology of cardiovascular diseases (CVD), with the goal in mind of contributing to a better understanding of their development, providing insights to guide the design of future, more personalized, medicine. As early as in 2001, CVD were recognized as the largest single cause of mortality on the planet [1]. Today, despite the huge growth of pathophysiology knowledge and advances in molecular mechanisms, prognosis, diagnosis, and treatment; CVD remains at the world’s top rank of all human death causes. In fact, the impact of CVD is growing and has reached pandemic proportions, mainly due to the great incidence of its comorbidities—obesity, diabetes and sedentary lifestyle, among others—in the global population [2].

Contributions to this Special Issue have been numerous, with eight original articles and four reviews being accepted for publication. These cover a wide molecular range of both vascular and cardiac pathophysiology very nicely, with an interesting focus on the implications of diabetes. There are three studies which base their major findings on comprehensive proteomic analyses, highlighting the huge technical capabilities of this approach [3,4,5]. Besides, two of the original articles offer interesting data supporting the therapeutical benefits of two hypoglycaemic drugs on left ventricular remodelling and cardiac dysfunction: the GLP-1 agonist DMB [5] and the sodium–glucose cotransporter 2 (SGLT2) inhibitor empagliflozin [6], shedding light on the implication of parkin-mediated mitophagy in their mechanism of action.

Endothelial relevance in the pathophysiology of CVD has been reinforced depicting an innovative link with calcification since one study has shown that poor endothelial function, led by nitric oxide (NO) impairment, contributes with classic pro-calcifying stimuli to trigger arterial calcification [3]. Furthermore, a systematic review on the impact of uremic toxins present with Chronic Kidney Disease on endothelial function, pinpoints an increase in inflammation, oxidative stress, leukocyte migration and adhesion, cell death and a thrombotic phenotype upon uremic conditions [7]. Additionally, an original article included in this issue has interestingly shown that unilateral acute renal ischemia-reperfusion (I/R) injury causes a cardiomyocyte Ca^2+^ cycle dysfunction, thereby impairing cardiac function [8]. These two contributions have brought the cardio-renal continuum into the hereby sketched molecular picture. Besides, a review publication has outlined the major mechanisms involved in Abdominal Aortic Aneurysm (AAA), a difficult-to-diagnose latent CVD, including inflammation, smooth muscle cells’ apoptosis, and the degradation of the extracellular matrix [9].

As the major outcome of vascular stenosis, due to underlying atherosclerosis within the coronary arteries, myocardial infarction (MI) is the major contributor to CVD-associated mortality and morbidity. Interestingly, the 1-year follow up of serum biomarkers after MI has shown an increase in the glial damage biomarker: glial fibrillary acidic protein (GFAP), but no changes in the neuronal damage biomarker: neurofilament light chain (NfL) [10]. Given that cognitive impairment after MI has been already described, this study suggests a pivotal contribution of glial cell damage in existing post-MI alterations of the central nervous system. A comprehensive vision of the clinical utility of classic and novel biomarkers of MI has been additionally included in the current Special Issue [11]. A protective role in the stress-induced cardiac remodelling of the dedicator of cytokinesis 10 (Dock10), a guanine exchange factors (GEF), has been also demonstrated [12], and paves the way for novel potential therapeutic interventions.

The contribution of diabetes mellitus (DM) to the pathophysiology of aortic valve stenosis (AS) has been thoroughly reviewed and evidences that diabetes has a detrimental effect on the quality of life and survival of AS patients [13]. Likewise, type 1 diabetes mellitus is hereby reported to impair rat cardiomyocyte contractility in the left and right ventricular free walls [14]. These two manuscripts have highlighted a substantial contribution of DM to the development and progression of heart dysfunction and show a potentially higher risk of heart failure in diabetic patients, led by mechanisms directly affecting the aortic valve and heart ventricles.

Altogether, this Special Issue adds substantial aspects to the molecular picture of arterial and cardiac stress-induced pathological processes, reinforcing key mechanisms, and outlining some new ones. Although the resulting picture is, certainly, far from becoming, some therapeutical hope arises from the novel targets described and the pharmacological interventions investigated.
